# The ETS Inhibitor YK-4-279 Suppresses Thyroid Cancer Progression Independent of *TERT* Promoter Mutations

**DOI:** 10.3389/fonc.2021.649323

**Published:** 2021-06-16

**Authors:** Junyu Xue, Shiyong Li, Peijie Shi, Mengke Chen, Shuang Yu, Shubin Hong, Yanbing Li, Rengyun Liu, Haipeng Xiao

**Affiliations:** ^1^ Department of Endocrinology, The First Affiliated Hospital, Sun Yat-sen University, Guangzhou, China; ^2^ Institute of Precision Medicine, The First Affiliated Hospital, Sun Yat-sen University, Guangzhou, China

**Keywords:** YK-4-279, telomerase reverse transcriptase, thyroid cancer, apoptosis, E-twenty-six transcription factor

## Abstract

Hotspot mutations in the core promoter region of the *telomerase reverse transcriptase* (*TERT*) gene have been well established to associate with aggressive clinical characteristics, radioiodine refractory, tumor recurrence, and mortality in thyroid cancer. Several E-twenty-six (ETS) transcription factors were reported to selectively bound to the mutant *TERT* promoter and activated TERT expression. In this study we aimed to investigate whether *TERT* promoter mutations confer sensitivity to ETS inhibitor YK-4-279 in thyroid cancer cells and whether this inhibitor could be served as a potential therapeutic agent for thyroid cancer. *In vitro* assays showed that YK-4-279 treatment sharply suppressed cell viability, colony formation, migration, and invasion, as well as induced cell cycle arrest and apoptosis in a panel of thyroid cancer cells. The cell viability after YK-4-279 treatment was similar between cell lines harboring mutant and wild-type *TERT* promoters. Furthermore, YK-4-279 treatment reduced both luciferase activity and mRNA expression of TERT independent of *TERT* promoter mutation status. Data from RNA-seq further revealed that YK-4-279 significantly affected biological processes including DNA replication and cell cycle. Reduced DNA helicase activity and decreased expression of several helicase genes were observed after YK-4-279 treatment. Moreover, YK-4-279 significantly inhibited tumor growth and induced apoptosis in a xenograft mice model. Thus, ETS inhibitor YK-4-279 suppressed TERT expression and conferred anti-tumor activity in a *TERT* promoter mutation-independent manner, and it could be a potential agent for the treatment of advanced thyroid cancers.

## Introduction

Thyroid cancer is the most prevalent endocrine cancer worldwide (1). Depending on its histological characteristics, thyroid cancer can be classified as papillary thyroid cancer (PTC), follicular thyroid cancer (FTC), anaplastic thyroid cancer (ATC), and medullary thyroid cancer (MTC). The MTC is derived from parafollicular C cells and accounts for approximately 4% of thyroid cancers (2), while the other three subtypes (PTC, FTC, and ATC) are all developed from follicular thyroid cells and account for the majority of thyroid cancers. Although most of the thyroid cancer cases have a good prognosis after standard clinical treatments, tumor recurrence and/or mortality still occurs in patients with aggressive thyroid cancer.

Genetic alterations drive the progression and contribute to the tumor recurrence and mortality of thyroid cancer (3). Molecular-based management of thyroid cancer is quickly developed in recent years and holds a great advantage to improve the survival of cancer patients with certain genetic alterations (4). *RET* mutations account for approximately 70% of MTC and associated with aggressiveness of thyroid cancer. Several RET-targeting inhibitors were developed and showed durable anti-tumor efficacy in RET-altered medullary thyroid cancer (5). *BRAF* V600E mutation, which constitutively activates MEK phosphorylation and MAPK/ERK signaling pathway, is the most prevalent genetic alteration in follicular-derived thyroid cancer, particularly in PTC (6, 7). Several inhibitors targeting *BRAF* mutation, such as vemurafenib and dabrafenib, have been discovered and show a dramatic response in advanced thyroid cancer patients harboring *BRAF* V600E mutation (8–10).

Hotspot mutations in the core promoter region of the *telomerase reverse telomerase* (*TERT*) gene are considered as the most frequent non-coding mutations in follicular-derived thyroid cancer (11). Numerous studies have demonstrated that *TERT* promoter mutations, especially when accompanied with *BRAF* V600E or *RAS* mutations, showed strong correlation to aggressive clinical characteristics, radioiodine refractory and poor clinical outcomes in patients with thyroid cancer (12–19). Several ETS factors, including GABPA, ETV1, ETV4, and ETV5 were reported to selectively bind to mutant *TERT* promoter and activate TERT expression (20–22). These findings raised the possibility that ETS inhibitors might be more sensitive to *TERT* promoter mutation-driven thyroid cancers. YK-4-279 is a well-described ETS factor inhibitor that has showed anti-tumor activity in Ewing’s sarcoma (23), prostate cancer (24), and neuroblastoma (25). Importantly, recent study showed that *BRAF*/*TERT* promoter double-mutated brain tumor cells were sensitive to YK-4-279 treatment, providing a therapeutic opportunity to manage brain tumor patients harboring both *BRAF* and *TERT* mutations (26). In this study we tested whether *BRAF* and/or *TERT* promoter mutations conferred sensitivity to YK-4-279 in thyroid cancer cells and whether this inhibitor could be served as a therapeutic agent for advanced thyroid cancers.

## Methods

### Cell Culture

The human thyroid cancer cell lines, including KTC-1, KHM-5M, Hth7, ACT1, CAL62, WRO, and TTA1 were obtained from the Type Culture Collection of the Chinese Academy of Sciences (Shanghai, China). Human thyroid cancer MDA-T41 was purchased from the American Type Culture Collection (ATCC, Manassas, VA, USA). The origins of human thyroid epithelial cell line Nthy-ori 3-1 and human PTC cell lines, TPC-1 and BCPAP, were as documented (27). BCPAP, KTC-1, KHM-5M, MDA-T41, and WRO were grown at 37°C in RPMI-1640 medium (Invitrogen, CA, USA) supplemented with 10% fetal bovine serum (FBS, #10270-106, Gibco, MD, USA). Nthy-ori3-1, TPC-1, ACT-1, CAL62, Hth7, and TTA1 were grown at 37°C in DMEM medium (Invitrogen, CA, USA) supplemented with 10% FBS.

### Inhibitor Preparation

The ETS inhibitor YK-4-279 (#S7679) was purchased from Selleck Chemicals (Houston, TX), dissolved in Dimethyl sulfoxide (DMSO) with a stock concentration of 10 mM and stored at −80°C. For *in vitro* study, YK-4-279 was used to treat cells for different concentrations as indicated for different experiments. For *in vivo* study, YK-4-279 was dissolved in DMSO to generated 15 mg/ml stocking solution and stored in −20°C. Working solution was dissolved as following proportion: 5% 15 mg/ml YK-4-279 solution + 40% PEG300 + 5% Tween 80 + 50% ddH_2_O. Vehicle was defined as working solution by replacing YK-4-279 with DMSO.

### RNA Extraction and Quantitative Real-Time PCR

Total RNA was extracted from cultured cells using the TRIzol reagent (#15596-018; Ambion, Life Technologies, Carlsbad, CA) and reverse-transcribed to cDNA using RevertAid First Strand cDNA Synthesis Kit (#K1622; ThermoFisher). Gene expression was analyzed in triplicate using PowerUp™ SYBR™ Green Master Mix (#A25742; Applied Biosystem) on the Applied Biosystems QuantStudio™ 7 Pro Real-Time PCR System. Relative expression of each gene was calculated according to the 2−ΔΔCt method. GAPDH was used as an internal control for normalization. The primers for TERT and GAPDH were used as described previously (20).

### Luciferase Reporter Assay

The luciferase reporter gene constructs containing the wild-type and mutant *TERT* promoters (pGL4-TERTp-WT, pGL4-TERTp-C250T and pGL4-TERTp-C228T) were obtained from Addgene (#84924, #84925, and #84926, respectively). For promoter activity assay, cells were seeded in triplicate into a 24-well plate and then transfected with 300 ng pGL4 plasmids containing the *TERT* promoter using Lipofectamine 3000 (Invitrogen) for 24 h followed by 1 μM of YK-4-279 treatment for 16 h. Rinella luciferase (pRL-TK) plasmid was transfected simultaneously with plasmid mentioned above as the normalizing control. After treatments, cells were lysed, and the luciferase activities were measured using the Dual-Luciferase Reporter Assay System (Promega). Relative luciferase activities were calculated by dividing firefly luciferase values with Rinella luciferase values for each set of reading.

### Cell Proliferation and Colony Formation Assays

For cell proliferation assay, cells (1,000–2,500 cells per well) were seeded on a 96-well plate, and Cell Counting Kit-8 (CCK-8) assay was carried out at day 5 to evaluate cell proliferation following the manufacturer’s instruction. Briefly, at the end of each culture period, 10 µl of the CCK-8 solution (#K1018, ApexBio) was added to each well. After incubation for 2 h, the absorbance was read at 450 nm. For colony-formation assay, 400 of KHM-5M and Hth7 cells or 1,000 of BCPAP cells were plated in duplicate in six-well plates. YK-4-279 treatment starts at 12 h after cell seeding. Culture media with YK-4-279 were replaced every 3 days. The colonies were photographed, and the total number of colonies ≥100 μm in diameter was counted after 2 weeks of culture.

### Cell Migration and Invasion Assays

To examine the effect of YK-4-279 on thyroid cancer migration and invasion, cell migration and invasion assays were performed in triplicates using Transwell^®^ chambers in 24-well plates following Ogasawara’s instruction (28). Transwell chambers with 8-μm pore polycarbonate membrane used for cell migration assay were obtained from Corning (Corning, NY). Chambers coated with Matrigel on the upper surface used for invasion assay were obtained from BD Biosciences (Franklin Lakes, NJ). Briefly, cells (5 × 10^4^ KHM-5M and Hth7 cells or 1.5 × 10^5^ BCPAP cells for migration assay; 1 × 10^5^ KHM-5M and Hth7 cells for invasion assay) were suspended in 250 μl of serum-free medium and placed in the upper chamber, while the lower chamber was loaded with 750 μl of cell culture medium with 10% FBS. Cells were incubated in 37°C with 5% CO_2_ for 6 h (migration) and 16 h (invasion), respectively. After incubation, the non-invaded cells were removed from the upper surface by a cotton swab. The invaded cells on the lower surface of the membrane were fixed in 4% paraformaldehyde for 15 min, washed three times with PBS, and stained with 0.5% crystal violet. Cells from three microscopic fields were photographed and counted.

### Cell Cycle and Apoptosis Analysis

For cell cycle analysis, experiments were performed using a commercial cell cycle and apoptosis detection kit (#40301ES60, Yeasen, China) following its instruction. Briefly, cells were collected and washed with PBS, fixed in 70% ethanol for 2 h at 4°C and then incubated in the staining solution with propidium iodide and RNase for 1 h. The apoptosis analysis was determined by the Pacific Blue™ Annexin V (#640918, San Diego, CA). Stained cells were detected by flow cytometry. Cell cycle distributions and the percentage of apoptotic cells were determined with FlowJo software.

### Caspase-3/7 Activity Detection

The activities of caspases-3 and caspase-7 were detected using the CellEvent™ Caspase 3/7 Green ReadyProbe™ Reagent (#R37111, Invitrogen) as previously reported (29). Briefly, cells were seeded on gelatin-coated slides in 24-well plate and treated with YK-4-279 at different doses (0, 0.3, and 1 µM) for 24 h. Cells were then incubated with 500 ul CellEvent™ Caspase 3/7 ReadyProbe™ Reagent dilution (1 drop/ml) for 30 min at 37°C followed by Hochest 33342 counterstaining for nuclei. Cells were then washed and fixed with 3.7% paraformaldehyde for 15 min and mounted with Anti-Fade Mounting media. Caspase-3/7 activated cells were observed under 200× magnificence with OLYMPUS IX83 Inverted Microscope (Olympus, Japan).

### RNA-Sequencing and Data Analysis

The cDNA libraries were constructed for each RNA sample using the TruSeq Stranded mRNA Library Prep Kit (Illumina, US) according to the manufacturer’s instructions. The mRNA library was prepared from total RNA using the NEBNext Small RNA Library Prep. Before read mapping, clean reads were obtained from the raw reads by removing the adaptor sequences and low-quality reads. The clean reads were then aligned to the human genome (GRCh38, NCBI) using Hisat2. HTseq was used to calculate gene counts, and the RPKM method was used to determine relative gene expression. Differential expression analysis of two conditions/groups (two biological replicates per condition) was performed using the DESeq2 R package (1.16.1). The resulting P-values were adjusted using the Benjamini and Hochberg’s approach for controlling the false discovery rate. Protein-coding genes larger than 1.5-fold alteration (0.587 for log_2_FC) with P-value <0.05 found by DESeq2 were defined Differential Expression Genes (DEGs) for further investigation in this study.

### Gene Ontology Analysis

Gene ontology (GO) analysis was performed to elucidate the biological implications of the differentially expressed genes identified in the experiment. We downloaded the GO annotations from NCBI (http://www.ncbi.nlm.nih.gov/), UniProt (http://www.uniprot.org/), and the Gene Ontology (http://www.geneontology.org/). Fisher’s exact test was applied to identify significantly enriched GO categories.

### Xenograft Tumorigenesis Assay

All animal experiments were approved and performed according to the guidelines of the Institutional Animal Care and Use Committee (IACUC) of Sun Yat-sen University. BALB/c nude mice (Female, 4–6 weeks old) were purchased from Vital River (Beijing, China) and housed in the SPF barrier facilities of Sun Yat-sen University. To ensure the similarity of xenograft tumor size, we followed the protocol of Yu lab described previously (30). Briefly, KHM-5M (1 × 10^7^) cells were injected subcutaneously into the axillary cavity of four-week-old nude mice and harvested when reaching 1 cm^3^. Necrotic area was dissected meanwhile the remaining compartment was fragmented as 1 mm^3^ pieces and transplanted into flanks of 6-week-old female nude mice (four mice for each group). When tumor sizes reached around 150 mm^3^, mice were treated daily with vehicle or 150 mg/kg of YK-4-279 by intraperitoneal injection. Tumor size was measured every day on the skin surface of the animal using a caliper, and tumor volume was calculated by the formula (L + W)^3^π/6. Mice were sacrificed when xenograft tumors in control group reached 1 cm^3^, and tumors were surgically removed, photographed, and weighted.

### Histological Analysis

The immunohistochemical staining was performed with primary antibodies on 4 μm-thick formalin-fixed paraffin-embedded tissue specimens as previously described (31). The primary antibodies used were as follows: Anti-Human Ki67 antibody (#ab92742, Abcam), anti-cleaved Caspase-3 antibody (#9664, Cell Signaling Technology). Semi-quantification analysis was performed by calculating percentage of positive staining cell under 200× magnification with the following formula: % of positive cell = counts of positive staining tumor nuclei/counts of all nuclei.

### Statistical Analysis

Three independent experiments were performed for all the *in vitro* assays, and each was done in triplicate. Results are reported as mean ± standard deviation (SD). The significance of differences between two groups was assessed by the Student’s t test. The multiple t-test was used to evaluate the difference of tendency of xenograft tumor growth. Statistical analyses were performed with GraphPad Prism v8.0. All the P values were two-sided and P <0.05 was considered as statistically significant.

## Results

### YK-4-279 Significantly Inhibited Thyroid Cancer Viability

**Figure 1:**
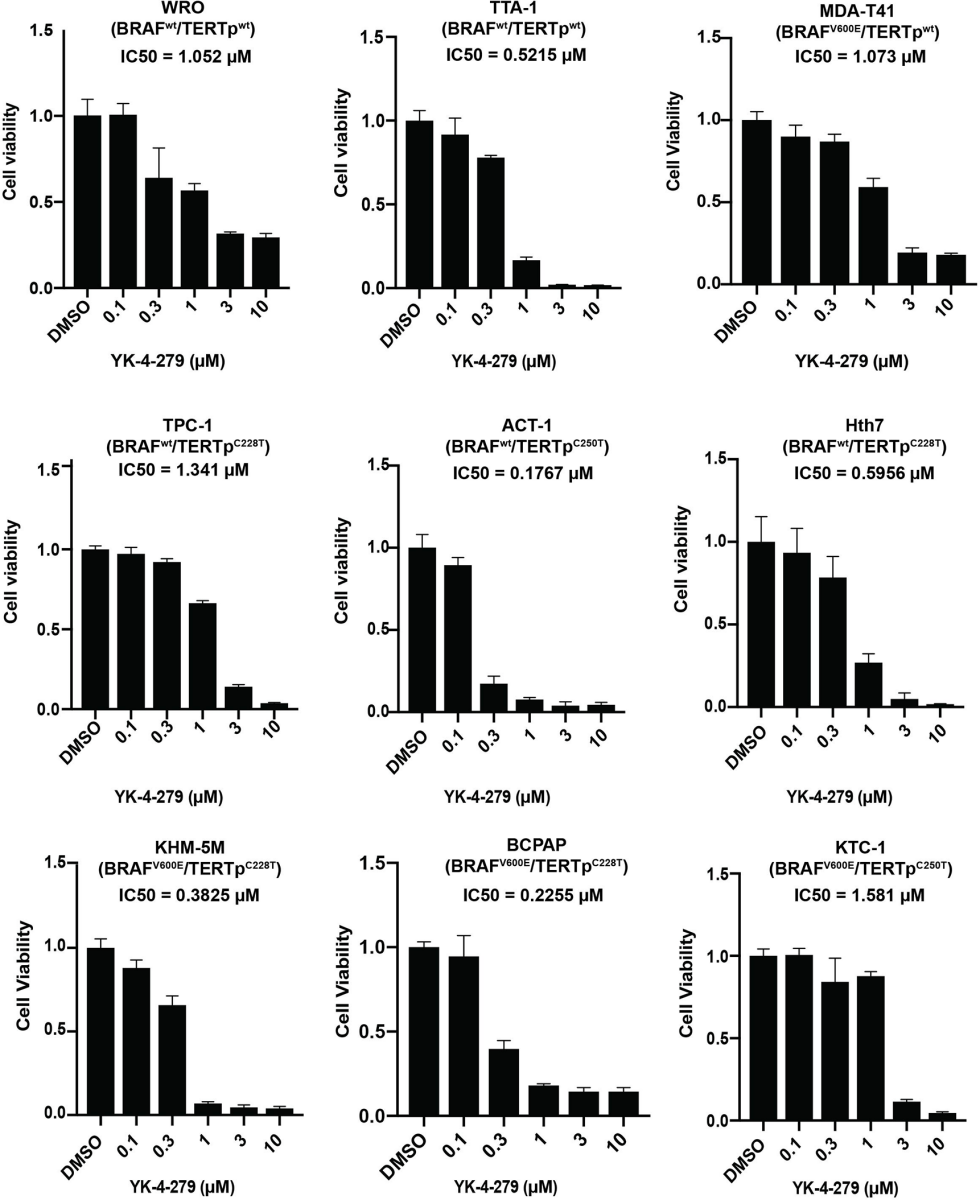
Effects of YK-4-279 on cell viability of thyroid cancer cells. Cells with different genetic backgrounds were treated by YK-4-279 with indicated concentrations for 5 days, followed by the CCK-8 assay. Drug with fresh medium was replenished every other day. Data represent relative cell growth at each indicated concentration of YK-4-279 in comparison with the control (0.1% DMSO). The results were shown as mean ± standard deviation (SD) of three independent experiments.

To investigate the effect of YK-4-279 on thyroid cancer, cell viability assay was first performed in nine thyroid cancer cell lines with different genetic backgrounds. As shown in **Figure 1**, treatment with 0.3 µM of YK-4-279 significantly inhibited the cell viability in most of the cell lines, and 3 or 10 µM of YK-4-279 almost abolished cell viabilities in all of the nine cell lines. The IC50 values for *BRAF*-mutant cell lines and *BRAF*-wild-type (WT) cell lines were 0.800 and 0.737 μM, respectively (P = 0.867); the IC50 values for *TERT* promoter mutation harboring cells and *TERT*-WT cells were 0.717 and 0.861 μM, respectively (P = 0.713). Moreover, there is no significant difference of the IC50 among the cell groups when we divided the cell lines into four groups based on the *BRAF* and *TERT* mutation status (**Supplementary Figure 1**). These data suggested that YK-4-279 suppressed thyroid cancer viability independent of *BRAF* V600E or *TERT* promoter mutations.

### YK-4-279 Suppressed TERT Expression in Thyroid Cancer Cells

**Figure 2:**
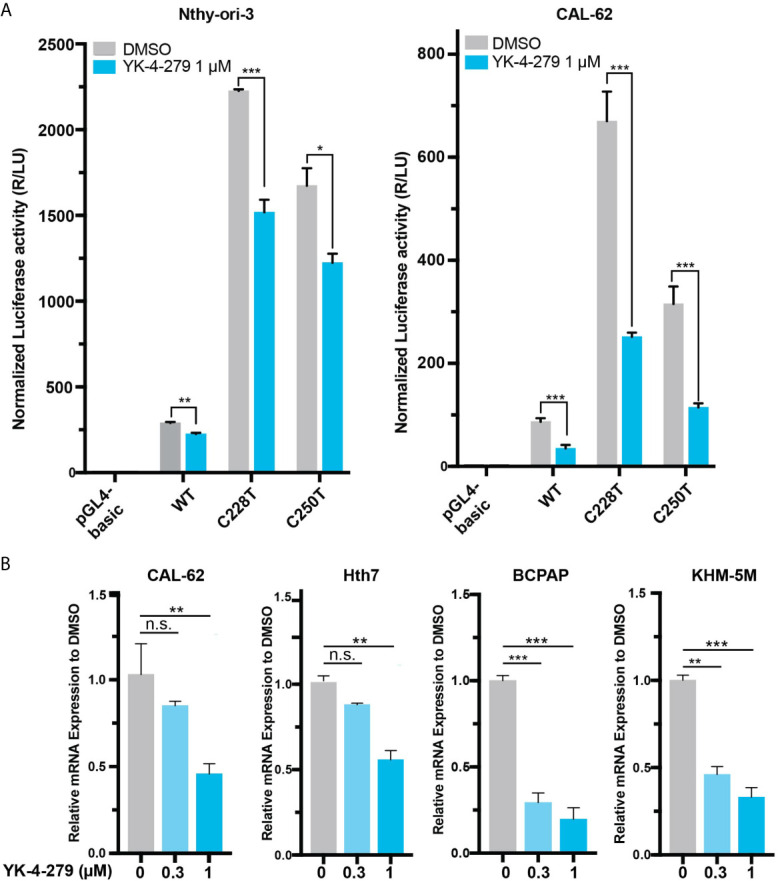
YK-4-249 regulates TERT expression in a *TERT* promoter mutation-independent manner. **(A)** YK-4-279 attenuated *TERT* promoter activities. Luciferase reporter plasmids carrying the wild-type (WT), C228T, or C250T mutant *TERT* promoters were transient transfected into Nthy-ori-3 and CAL62 cells, and then the cells were treated with 1 μM of YK-4-279 for 16  h, followed by luciferase reporter assay. **(B)** YK-4-279 treatment inhibited the mRNA expression of *TERT*. Four cancer cell lines harboring either *BRAF* V600E and/or *TERT* promoter mutation were treated with 1 μm of YK-4-279 for 24 h, followed by qRT-PCR for *TERT* mRNA expression detection. *P < 0.05, **P < 0.01, ***P < 0.001, by two-tailed Student’s t test. n.s., not significant. All the values represent the average ± standard deviation (SD) of triplicate samples from a typical experiment. Similar results were obtained in two additional independent experiments.

Since TERT is a master regulator in human cancer and plays vital roles in thyroid cancer, we hypothesized that the effect of YK-4-279 on thyroid cancer viability may be mediated, at least partially, by regulating TERT expression. To address this, we treated cells with YK-4-279 and tested the promoter activity and mRNA expression of TERT in thyroid cells. Luciferase assay showed that YK-4-279 reduced luciferase activities in thyroid cells transfected with wild-type, C228T, and C250T-mutant *TERT* promoter (**Figure 2A**). Consistently, treatment of thyroid cancer cells with YK-4-279 for 24 h significantly decreased TERT expression in either *TERT* promoter-WT or *TERT* promoter-mutant cell lines in a dose-dependent manner (**Figure 2B**). These results indicated that YK-4-279 regulated *TERT* transcription in a *TERT* promoter mutation-independent manner.

### YK-4-279 Treatment Attenuated the Aggressive Characteristics of Thyroid Cancer

**Figure 3:**
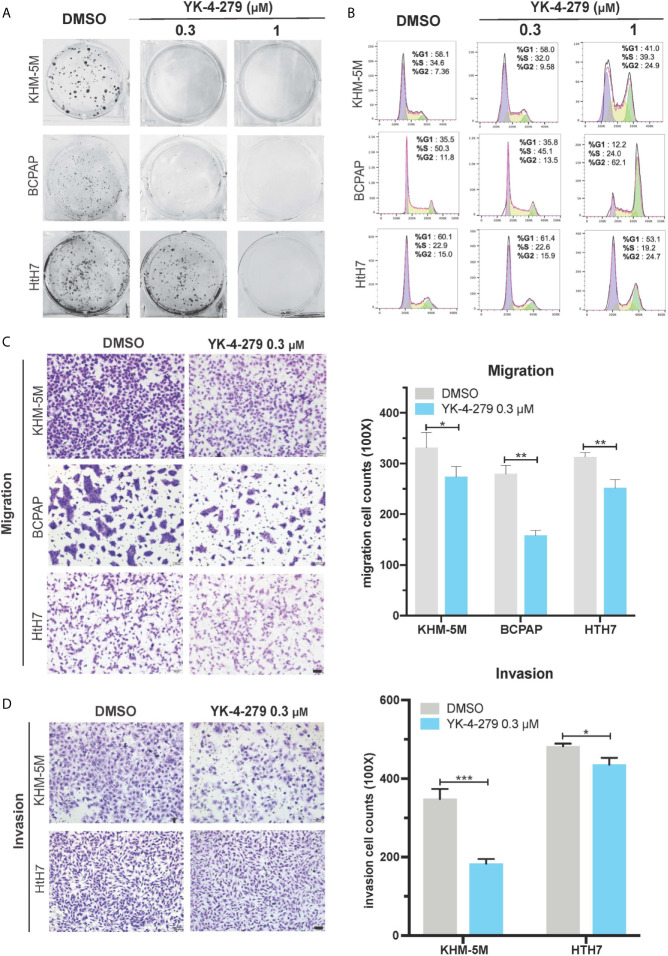
Effects of YK-4-279 on the oncogenic behaviors of thyroid cancer cells. Thyroid cancer cells were treated with DMSO or YK-4-279, followed by performance of monolayer colony formation **(A)**, cell cycle analysis **(B)**, Transwell cell migration **(C)** and cell invasion assays **(D)**. “DMSO” in **(A−D)** represented 0.1% of DMSO in fresh culture media. The scale bars in panels **(C**, **D)** represent 100 μm. *P < 0.05, **P < 0.01, ***P < 0.001, by two-tailed Student’s t test. All the values represent the average ± standard deviation (SD) of triplicate samples from a typical experiment. Similar results were obtained in two additional independent experiments.

To comprehensively investigate the effects of YK-4-279 on aggressive behaviors of thyroid cancer cells, we next used *in vitro* models to examine the roles of YK-4-279 in oncogenic behaviors of aggressive thyroid cancer cell lines harboring both *BRAF* V600E and *TERT* promoter mutations (BCPAP and KHM-5M) or harboring *TERT* promoter mutation only (Hth7). As shown in **Figure 3A**, YK-4-279 inhibited anchorage-dependent survival of BCPAP, KHM-5M and Hth7 in plain six-well plates in a dose-dependent manner. Cell cycle is another important parameter for cell proliferation and survival. Previous papers indicated that YK-4-279 impeded cell proliferation *via* G2/M arrest in Ewing’s sarcoma (32) and neuroblastoma (25). We further investigated the role of YK-4-279 on cell cycle *via* flow cytometry. After administration of YK-4-279 for 16 h, significantly increased G2/M proportion was observed when treated with 1 μM of YK-4-279 in all cell lines that we tested, showing that YK-4-279 induced G2/M arrest in thyroid cell lines (**Figure 3B**).

In addition to cell proliferation and survival, migration and invasion are crucial properties of cancer aggressiveness. Since no significant increase of G2/M proportion of cell cycle in thyroid cancer cell lines was observed after 16 h of YK-4-279 treatment at 0.3 μM (**Figure 3B**), we used this concentration to investigate the effect of cell migration and invasion of thyroid cancer *via* Boyden chamber with or without Matrigel, respectively. Our data showed that 0.3 μM of YK-4-279 significantly reduced cell migration and invasion in all tested cell lines (**Figures 3C, D** **)**. Thus, we confirmed that YK-4-279 displayed regressive role on the oncogenic behaviors of thyroid cancer in a dose-dependent manner. Taken together, these data demonstrated that YK-4-279 significantly suppressed aggressive characteristics of thyroid cancer cells *in vitro*.

### YK-4-279 Induced Apoptosis in Thyroid Cancer Cells

**Figure 4:**
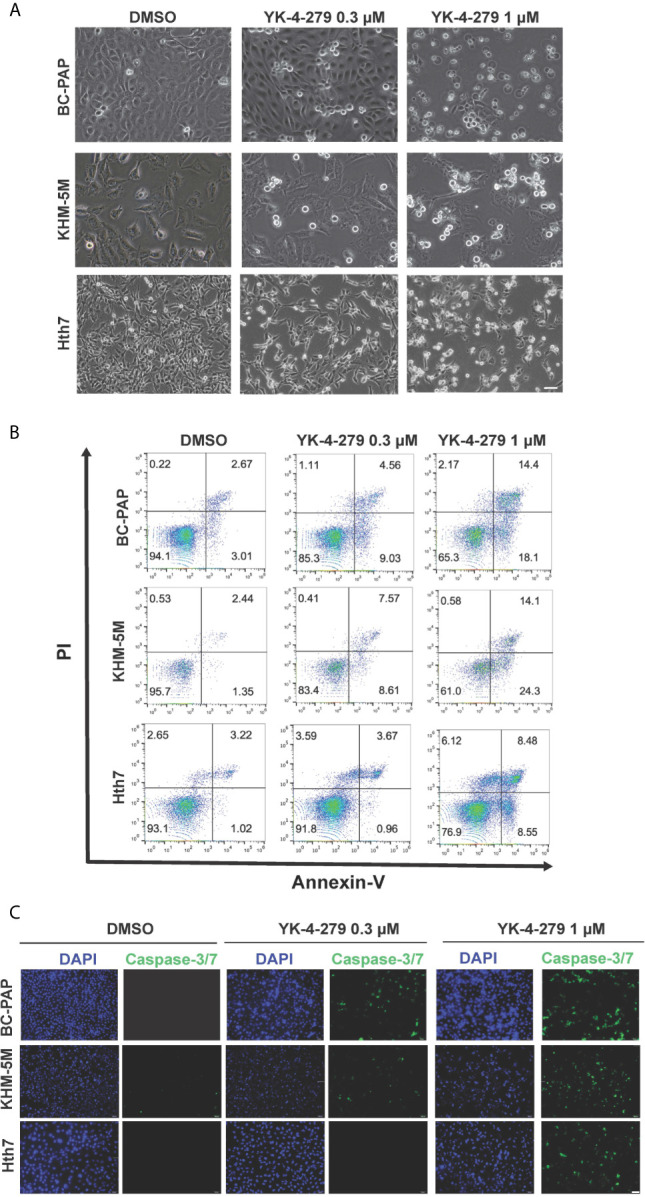
YK-4-279 induces cell apoptosis in thyroid cancer cells. Thyroid cancer cells (KHM-5M, BCPAP, and Hth7) were treated with DMSO or different dosages of YK-4-279 for 24 h. **(A)** Cell morphological changes (200×) after DMSO or YK-4-279 treatment. **(B)** Flow cytometry analysis of apoptosis by PI/Annexin-V staining. **(C)** Examination of caspase-3/7 activity after DMSO or drug treatment. The scale bars in panels **(A, C)** represent 100 μm. Similar results were obtained in two additional independent experiments.

After treating thyroid cancer cells with YK-4-279 at different concentrations (0, 0.3, and 1 μM) for 24 h, increased floating apoptotic cells with round-shaped morphology were observed (**Figure 4A**), indicating that YK-4-279 may induce apoptosis in these cells. To confirm this, we performed PI/Annexin-V double staining assay in YK-4-279 treated cell lines and detected the apoptotic cells by flow cytometry. As a result, we found that the proportions of apoptotic (Annexin V-positive) cells were increased by four to nine times after treatment by 1 μM of YK-4-279 compared to the DMSO groups (**Figure 4B**, **Supplementary Figure 2**). Furthermore, we examined activity of caspase-3/7, two major apoptosis-related effector, in treated thyroid cell lines and observed a dose-dependent enhancement of caspase-3/7 activity in all tested cell lines (**Figure 4C**). These results showed that YK-4-279 induced cell apoptosis in thyroid cancer cells.

### Putative Targets for YK-4-279 Treatment

**Figure 5:**
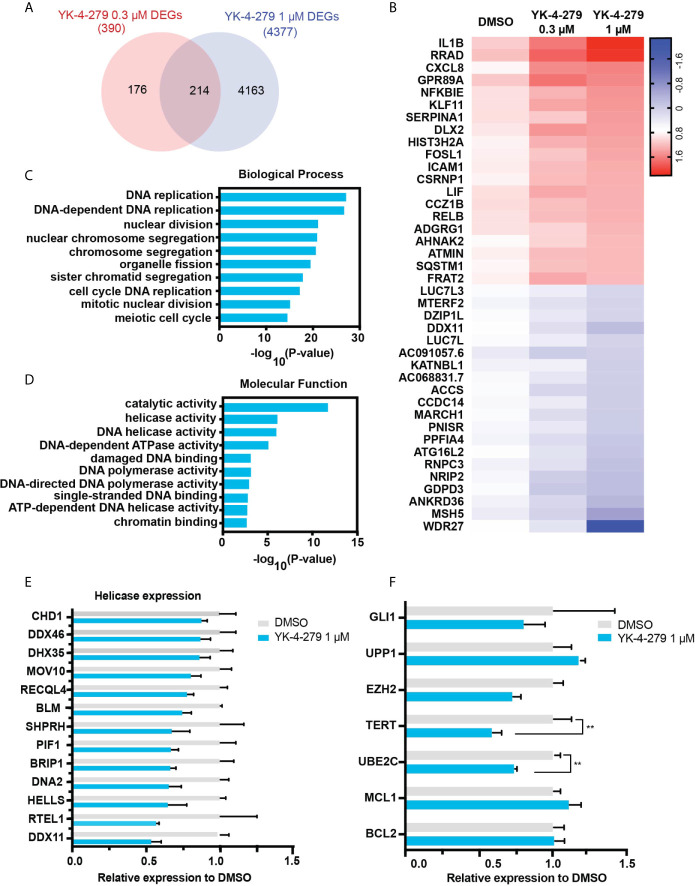
RNA-seq analysis for YK-4-279 treatment. **(A)** Differentially expressed genes (DEGs) in YK-4-279 treated KHM-5M cells compared with cells treated with DMSO. **(B)** Representative does-dependent DEGs. Blue referred to downregulated DEGs whereas Red referred to upregulated DEGs. **(C, D)** Gene ontology enrichment for biological process and molecular function analysis. **(E)** Significant downregulated DNA helicase in KHM-5M cells treated with 1 μM of YK-4-279 compared to the DMSO group. **(F)** mRNA expression of selected genes after DMSO or YK-4-279 treatment. ** refers to P < 0.01 by two-tailed Student’s t test.

To systematically explore gene expression profile and pathway affected by YK-4-279, RNA-seq was performed in KHM-5M cells treated with DMSO or YK-4-279 at different dosages (0.3 and 1 μM). Compared with the control group, 390 and 4,377 differentially expressed genes (DEGs) were identified in KHM-5M cells treated with 0.3 and 1 μM of YK-4-279, respectively. Furthermore, 214 DEGs were overlapped in both two treated groups (**Figure 5A**). The top 20 upregulated and downregulated DEGs were presented in **Figure 5B**. To interpret the crucial pathways affected by YK-4-279, gene ontology (GO) enrichment analysis based on the DEGs was performed. Results showed that the YK-4-279 treatment was significantly associated with biological processes including DNA replication, nuclear division, chromosome segregation, and meiotic cell cycle (**Figure 5C**). In addition, molecular function enrichment analysis revealed that catalytic activity, DNA helicase activity, DNA-dependent ATPase activity, and ATP-dependent DNA helicase activity were remarkably correlated with YK-4-279 (**Figure 5D**). Consistently, a number of DNA helicase genes were significantly downregulated after YK-4-279 treatment (**Figure 5E**), indicating a potentially pivotal role of DNA helicase activity in mediating the effect of YK-4-279 in thyroid cancer. Moreover, we checked the expression of several genes that were previously reported to be affected by YK-4-279 in Ewing’s sarcoma (32, 33) and found that the expressions of TERT and UBE2C (Ubiquitin Conjugating Enzyme E2-C) were reduced after YK-4-279 treatment in our cell lines (**Figure 5F**). The expressions of each ETS transcriptional factor were not changed in KHM-5M cells after treatment with YK-4-279 (**Supplementary Figure 3**).

### YK-4-279 Reduced Tumorigenesis in Xenograft Mouse Model

**Figure 6:**
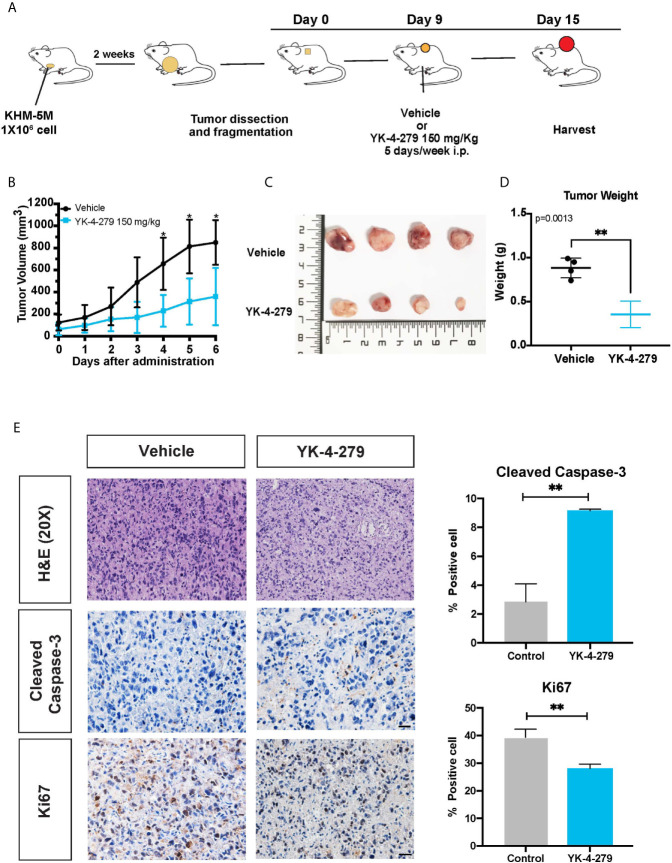
YK-4-279 inhibits *in vivo* tumor growth. **(A)** Diagram for the xenograft mice model in this study. **(B)** The tumor growth curves of xenograft model. **(C)** Photograph of the xenograft tumors (n = 4). The numbers on ruler refer to centimeter. **(D)** Weights of xenograft tumors. **(E)** Representative figures of H&E staining and immunohistochemistry for cleaved caspase-3 and Ki-67. Scale bar, 100 μm. Semi-quantification of immunohistochemistry staining was performed by calculating the percentage of positive staining cell. * refers to P < 0.05, ** refers to P < 0.01, by two-tailed Student’s t test. Triplicated experiments were performed with similar results obtained.

To determine whether the anti-cancer effects of YK-4-279 observed for thyroid cancer cells was re-capitulated *in vivo*, a xenograft mouse model was established and treated with YK-4-279 by intraperitoneal injection (**Figure 6A**). As shown in **Figures 6B, C**, daily treatment with YK-4-279 at dosage of 150 mg/kg significantly suppressed the growth of xenograft tumors. Consistently, tumor weights in YK-4-279 treatment group were remarkably lower than those in the control group (**Figure 6D**). Along with the growth suppression, as shown in **Figure 6E**, immunohistochemistry of xenograft tumors showed that the expression of proliferation marker, Ki-67, was decreased; while the apoptosis marker, cleaved caspase-3, was increased in YK-4-279 treatment group compared to that in controls. Morphological feature of apoptosis was also observed by H&E staining in the drug treated tissue section (**Figure 6E**). These data showed that YK-4-279 was capable of reducing thyroid tumorigenesis *in vivo*.

## Discussion

The anti-tumor activity of YK-4-279 was first reported in Ewing’s sarcoma and then confirmed by studies performed in several cancer types, including prostate cancer, neuroblastoma, lymphoma, and glioma (23–26, 34). This is the first study, to the best of our knowledge, to investigate the effect of YK-4-279 on thyroid cancer cells, and our data showed a significant inhibitory effect on the aggressive characteristics of thyroid cancer. Specifically, YK-4-279 treatment sharply suppressed cell growth, survival, migration, and invasion of thyroid cancer cells, and it induced G2/M cell cycle arrest and apoptosis in all the thyroid cancer cell lines we tested. Importantly, our data showed that YK-4-279 robustly suppressed tumor growth in a xenograft thyroid tumor model. These results demonstrated that YK-4-279 efficiently inhibited tumor growth and progression of thyroid cancer and exerted therapeutic potential for aggressive thyroid cancer management.

YK-4-279 was initially reported as a small molecule targeting the interaction of oncogenic protein EWS-FLI1 with RNA helicases DHX9 and DDX5 in Ewing’s sarcoma (23). Recently, several papers revealed that YK-4-279 exerted similar tumor-inhibitory effect in cancer cell lines within overexpression of other ETS-factors. Rahim et al. reported that YK-4-279 specifically reduced tumor growth and metastasis of ETV1-fusion positive prostate cancers by reducing ETV1 transcriptional activity (35). Similarly, exposure to TK-216, the clinical derivative of YK-4-279, reduced the interaction of ETS factors SPIB and SPI1 with these RNA helicases in B-cell lymphomas (34). Furthermore, YK-4-279 showed powerful inhibition of tumor proliferation and mitosis in ETS-FLI1 negative neuroblastoma, suggesting that YK-4-279 might disrupt protein interactions required for mitosis and proliferation (25). These data indicated that novel ETS factor-related mechanism may be involved in YK-4-279 induced solid tumor inhibition.

Several ETS factors, including ETS1, ETS2, ELK1, ELF3, ETV5, and EHF have been shown to be overexpressed during thyroid carcinogenesis, and inhibition of these ETS factors constrained cell proliferation, colony formation, migration, invasion and induced cell cycle arrest and apoptosis (36–42), which was consistent with what we observed after YK-4-279 treatment. Importantly, some classical oncogenes, such as PIK3CA, TWIST1, HER2, and HER3 were identified as the direct targets of ETS factors in thyroid cancer (40–42). Considering that the expression of each ETS factor was not changed after YK-4-279 treatment, the anti-tumor effect of YK-4-279 on thyroid cancer might be implemented by preventing the binding of certain ETS factors to their targets.

To comprehensively identify putative targets and pathway response to YK-4-279 treatment in thyroid cancer, RNA-profiling was performed in the present study. We found that DNA helicase activities were remarkably enriched in GO analysis, while decreased expression of several known DNA helicases was observed after treatment with YK-4-279 in our RNA-seq expression data. These results suggested that DNA helicases are likely to be the key targets and mediators of YK-4-279 treatment in thyroid cancer. We postulated that YK-4-279 may directly impede ETS factor binding to the promoter regions of DNA helicase genes since duplicate ETS motifs were identified as essential transcription regulatory elements in the promoters of multiple DNA helicase-encoding genes (43). However, the essential mechanism of YK-4-279 on DNA helicase regulation required to be further addressed.

Telomerase reverse transcriptase (TERT) is the catalytic component of the telomerase complex, which plays a key role in maintaining telomere length and cell immortality and in controlling cellular activities. Activation of TERT expression promoted cell proliferation without telomere shortening and was linked to cancer hallmark behaviors in several solid cancers (44). Inhibition of TERT expression in thyroid cancer cells significantly reduced cell viability, migration, and invasion *in vitro* and suppressed tumor growth *in vivo* (20, 45, 46). In the present study, we found that YK-4-279 treatment reduced TERT expression and the cells treated by YK-4-279 showed similar phenotypes with TERT silencing cells. Therefore, in addition to regulating DNA helicase expression, the anti-tumor activity of YK-4-279 can be explained partially by suppressing the oncogenic effect of TERT in thyroid cancer.

The two hotspot mutations in *TERT* promoter were frequently observed in patient with PTC, FTC, PDTC, and ATC, but not in MTC (11, 47). The *BRAF* V600E and the MAPK pathway was reported to activate several ETS transcription factors, the latter then selectively bonded to the mutant promoter of *TERT* and led to TERT activation in thyroid cancer (20–22). In contrast to the previous findings that *BRAF*/*TERT* promoter double-mutated brain tumor cells (26) were hypersensitive against the ETS inhibitor YK-4-279, we found that YK-4-279 reduced the expression of TERT and conferred the anti-tumor activity independent of *TERT* promoter mutations in thyroid cancer cells. This discrepancy between brain tumors and thyroid cancer can be explained by alternative mechanism of ETS factors on TERT regulation among these cancer types. Takahashi and his colleagues reported that EWSR1–ETS fusion activated hTERT expression in Ewing’s sarcoma by recruiting p300 to *TERT* promoter in an ETS factor binding site-independent manner (48). In thyroid cancer, phosphorylation and activation of ETS factor ELK1 by BRAF/MAPK pathway were necessary for thyroid-specific FOXE1 recruitment to *TERT* promoter *via* direct ELK1–FOXE1 interaction and this recruitment was independent from cis-acting mutations of *TERT* promoter (49). Thus, it is likely to the case that the interaction between ETS and certain transcription activators, such as FOXE1, play an important role in TERT activation in thyroid cancer cells while YK-4-279 inhibits TERT expression by disrupting ETS factors’ interaction. Further investigation on the mechanism of YK-4-279 on ETS recruitment of co-activators on *TERT* promoter in thyroid cancer is required.

In conclusion, our results demonstrate that YK-4-279 is powerful in arresting cell cycle and inducing apoptosis, leading to decreased cell growth and progression of thyroid cancer. The anti-tumor activity of YK-4-279 in thyroid cancer is independent of *TERT* promoter mutations and likely to be explained by inhibiting the expression of *TERT* and several DNA helicase genes. Although the exact mechanism needs to be further investigated, YK-4-279 is a promising therapeutic agent for the treatment of aggressive thyroid cancers.

## Data Availability Statement

The datasets presented in this study can be found in online repositories. The names of the repository/repositories and accession number(s) can be found below: GEO repository and accession GSE171473 https://www.ncbi.nlm.nih.gov/geo/query/acc.cgi?acc=GSE171473.

## Ethics Statement

The animal study was reviewed and approved by the Institutional Animal Care and Use Committee (IACUC) of Sun Yat-sen University.

## Author Contributions

HX and RL designed the study. JX, SL, PS, MC, SY, and SH carried out the experiments. JX, SL, and RL contributed to data analysis. JX prepared the figures and drafted the manuscript under the supervision of RL, YL, and HX. All authors contributed to the article and approved the submitted version.

## Funding

This study was supported by grants from the National Natural Science Foundation of China (No. 81772850 and No. 82072952), the Guangzhou Science and Technology Project (No. 201803010057) and China Postdoctoral Science Foundation (No. 2020M683125).

## Conflict of Interest

The authors declare that the research was conducted in the absence of any commercial or financial relationships that could be construed as a potential conflict of interest.
