# Supplementary material for: The ETS Inhibitor YK-4-279 Suppresses Thyroid Cancer Progression Independent of TERT Promoter Mutations
Source: Front Oncol. 2021 Jun 16;11:649323. doi: 10.3389/fonc.2021.649323 (PMC8242932; doi:10.3389/fonc.2021.649323)
Supplement: Supplementary file 1 [file DataSheet_1.pdf]

Supplimentary Figure 1

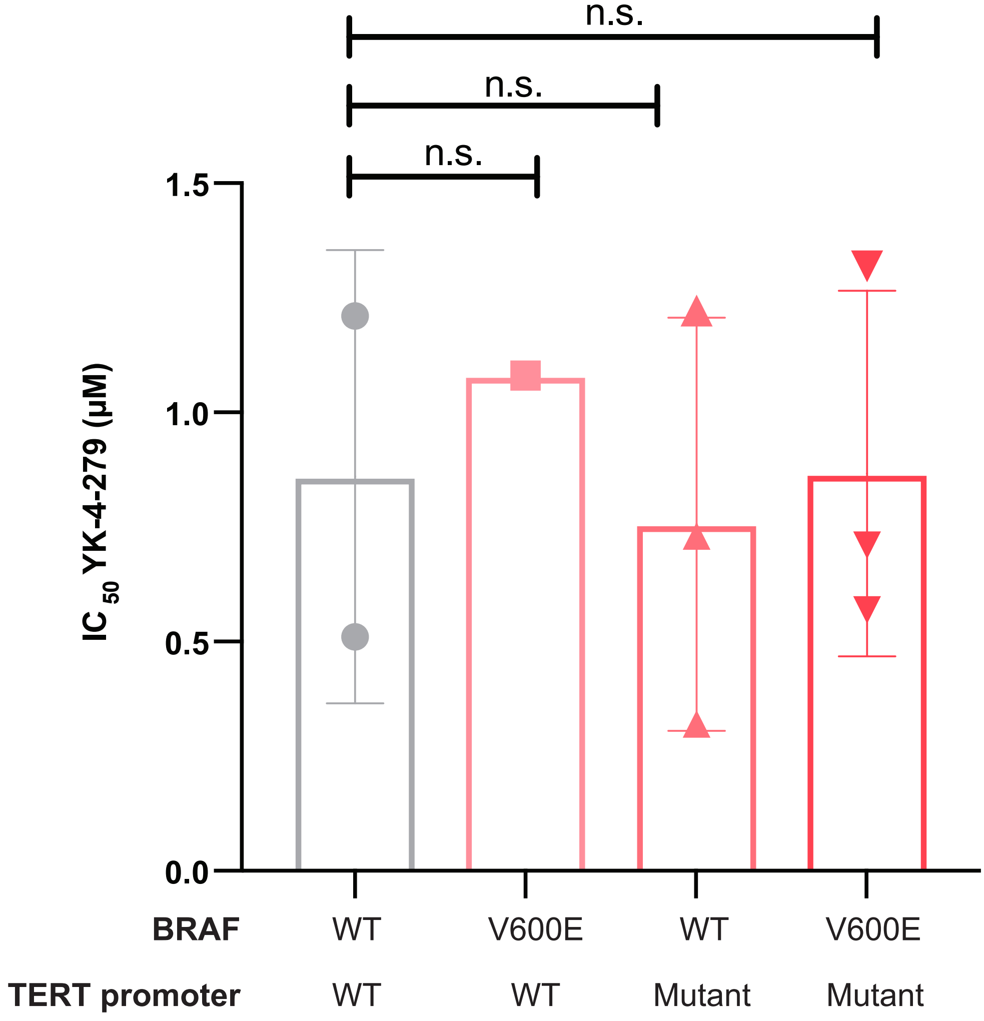

Supplementary Figure 1. Comparation of IC50s between different groups of thyroid cancer cell lines. n.s., not significant.

Supplementary Figure 2

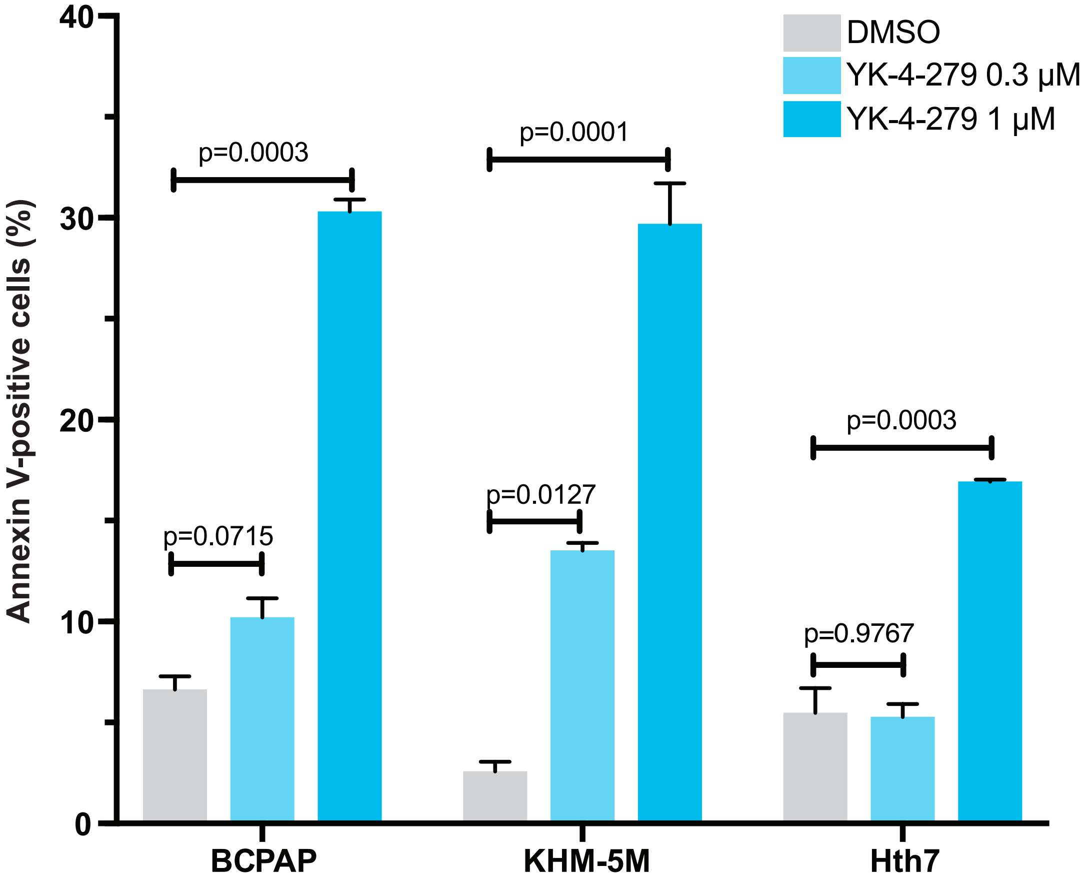

Supplementary Figure 2. The percentage of Annexin V-positive cells after treatment with YK-4-279. All the values represent the average ± standard deviation (SD) of triplicate samples from a typical experiment. P values were calculated by two-tailed Student's t test. Similar results were obtained in two additional independent experiments.

Supplimentary Figure 3

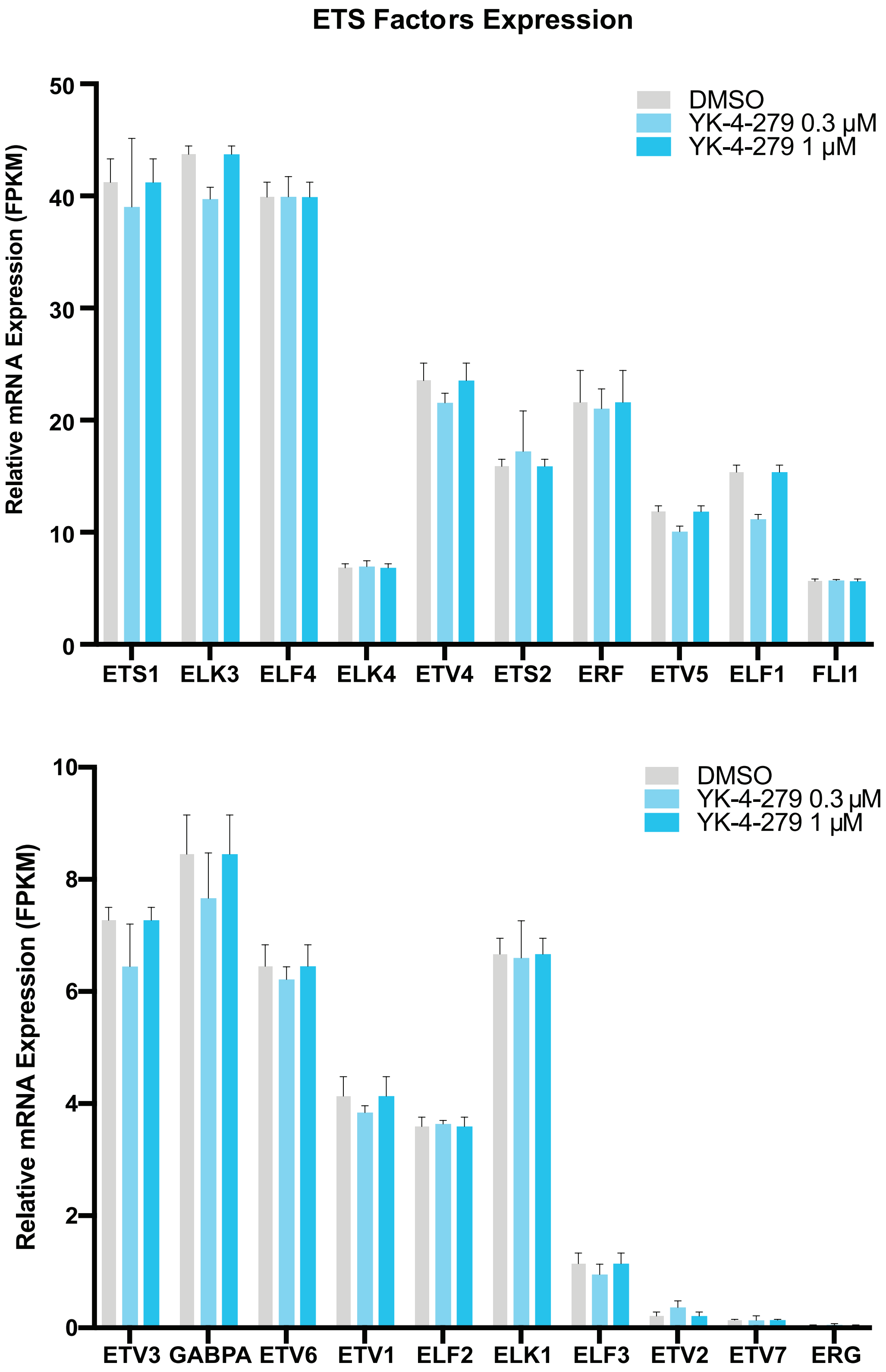

Supplementary Figure 3. The expression of each ETS transcription factors in KHM-5M cells after YK-4-279 treatment. All the values represent the average ± standard deviation (SD) of triplicate samples.
